# AmazonForest: In Silico Metaprediction of Pathogenic Variants

**DOI:** 10.3390/biology11040538

**Published:** 2022-03-31

**Authors:** Helber Gonzales Almeida Palheta, Wanderson Gonçalves Gonçalves, Leonardo Miranda Brito, Arthur Ribeiro dos Santos, Marlon dos Reis Matsumoto, Ândrea Ribeiro-dos-Santos, Gilderlanio Santana de Araújo

**Affiliations:** 1 Laboratory of Human and Medical Genetics, Graduate Program of Genetics and Molecular Biology, Institute of Biological Sciences, Federal University of Pará, Belém 66075-110, Brazil; hpalheta@gmail.com (H.G.A.P.); wandersongegoncalves@gmail.com (W.G.G.); lb9458@gmail.com (L.M.B.); arthurrdsantos@outlook.com (A.R.d.S.); marlonmatsumotosdb@gmail.com (M.d.R.M.); akelyufpa@gmail.com (Â.R.-d.-S.); 2 Research Center on Oncology, Graduate Program of Oncology and Medical Science, Federal University of Pará, Belém 66073-000, Brazil

**Keywords:** metaprediction, encoding data, random forest, representation learning, genetic variants, clinical impact, functional impact

## Abstract

**Simple Summary:**

ClinVar is a valuable platform that stores a large set of relevant genetic associations with complex phenotypes. However, the functional impact of a partial set of such associations remains misinterpreted, due to the presence of variants with uncertain significance or with conflicting pathogenicity interpretations. To fill this gap, we present AmazonForest: a metaprediction model based on Random Forest for pathogenicity prediction. AmazonForest was used to reclassify a set of ∼101,000 variants that were predicted as having high pathogenic probability. AmazonForest is available as a web tool with a simple web interface, and also as an R object for pathogenicity predictions.

**Abstract:**

ClinVar is a web platform that stores ∼789,000 genetic associations with complex diseases. A partial set of these cataloged genetic associations has challenged clinicians and geneticists, often leading to conflicting interpretations or uncertain clinical impact significance. In this study, we addressed the (re)classification of genetic variants by AmazonForest, which is a random-forest-based pathogenicity metaprediction model that works by combining functional impact data from eight prediction tools. We evaluated the performance of representation learning algorithms such as autoencoders to propose a better strategy. All metaprediction models were trained with ClinVar data, and genetic variants were annotated with eight functional impact predictors cataloged with SnpEff/SnpSift. AmazonForest implements the best random forest model with a one hot data-encoding strategy, which shows an Area Under ROC Curve of ≥0.93. AmazonForest was employed for pathogenicity prediction of a set of ∼101,000 genetic variants of uncertain significance or conflict of interpretation. Our findings revealed ∼24,000 variants with high pathogenic probability ($RF_{prob}\geq 0.9$). In addition, we show results for Alzheimer’s Disease as a demonstration of its application in clinical interpretation of genetic variants in complex diseases. Lastly, AmazonForest is available as a web tool and R object that can be loaded to perform pathogenicity predictions.

## 1. Introduction

Next-generation sequencing (NGS) methods have allowed whole-genome analyses for humans and other species. Genome-wide association studies (GWAS) and candidate gene studies have produced a large volume of genetic associations between single-nucleotide polymorphisms (SNPs) and insertions/deletions (INDELs) with complex diseases. Most of these associations show variable effects and genetic diversity among populations [1,2]. Variants with highly pathogenic effects are responsible for developing several types of cancer [3], Type 2 diabetes [4], and Alzheimer’s disease [5,6]. Understanding the biological role and impact of these variants on clinical and personalized levels is a complex task.

ClinVar is an online database that stores around 789,000 curated entries that show associations between phenotypes and genetic variants (SNPs or INDELs) and their clinical relevance (classified as either benign or pathogenic) [7]. ClinVar has improved our understanding of the functional role of genetic variants as research increasingly focuses on precision medicine [8]. However, many genetic variants are functionally misinterpreted and continue to have conflicting interpretations (CI) or uncertain significance (VUS).

Distinct machine learning (ML) metaprediction models have been proposed for pathogenicity prediction of genetic variants, aiming to combine the strengths of multiple pathogenicity prediction programs. Each metaprediction model has been suggested for the analysis of a single variant class (synonymous or nonsynonymous variants) [9,10,11,12,13], and most metapredictors were used for the pathogenicity prediction of VUS and CI variants [9,10,12,13]. Interestingly, most recently proposed metapredictors are decision tree-based or an ensemble of decision trees, which constitute models with clear interpretations. Ensemble-based methods, such as Random Forest (RF), are promising for pathogenicity prediction of coding and noncoding variants [9,10,11,13]. However, these models have shown differences regarding data-training methods, specifically on data heterogeneity and on the number of features used to train and test each classification model.

Thus, we implemented AmazonForest, a pathogenicity metapredictor based on Random Forest and functional impact data for high confidence pathogenicity interpretation. AmazonForest is the main contribution of this work. In addition, we employed the AmazonForest model to reclassify 100,805 genetic variants, and make available a dataset of ∼24,000 genetic variants with high pathogenic probability ($RF_{prob}$ ≥ 0.9). The resulting dataset sums as a large collection of annotated potentially disease-causing variants that may aid in the investigation and modeling of diseases.

## 2. Materials and Methods

### 2.1. Fetch ClinVar .vcf File

The first step consists of fetching genome-wide and clinical data from ClinVar, which is stored in .vcf files. The .vcf file is available at https://ftp.ncbi.nlm.nih.gov/pub/clinvar/vcf_GRCh38/, accessed on 2 February 2021. The dataset showed 789,419 genetic variants. Each variant is classified according to the ACMG-AMP [14] with labels that correspond to the following categories: benign, likely benign, variant of uncertain significance, likely pathogenic, pathogenic, or conflict of interpretation.

### 2.2. Functional Impact Variant Annotation by Single Predictors

SnpEff and SnpSift (v.4.3) configured with dbNSFP4.0 were used for functional annotation of variants stored in ClinVar .vcf files. Therefore, our metapredictor was built based on categorical data extracted from eight predictors: FATHMM, SIFT, PolyPhen-2 HVAR, PolyPhen-2 HDIV, PROVEAN, MutationAssessor, MutationTaster2, and LRT. Each predictor is independent and based on distinct genomic approaches such as sequence characteristics, conservation, and amino acid changes. All predictors are described in detail as follows:

- FATHMM predicts the functional effects of coding and noncoding variants. This predictor combines wild-type and mutated sequences in a hidden Markov model, which identifies mutations in peptide chains, showing the alignment of homologous sequences and conserved protein domains [15];
- SIFT (Sorting Intolerant From Tolerant) is a prediction tool that codes an algorithm for amino acid substitution analyses. It assumes that important positions in a protein sequence have been conserved throughout evolution, and therefore substitutions at these positions may affect protein function. The algorithm sorts changes in a polypeptide chain as tolerant or intolerant according to its evolutionary conservation [16];
- Polyphen-2 (Polymorphism Phenotyping v2) predicts the impact of amino acid substitutions on structural stability, physical interactions, and human protein function. The probability of a mutation being pathogenic depends on the extraction of sequence annotations, structural attributes, and conservation profiles in protein-coding regions [17];
- PROVEAN (Protein Variation Effect Analyzer) is a predictor that provides a generalized approach to predict the functional effects on variations in a peptide chain. These effects include SNPs, INDELs, or multiple amino acid substitutions. Prediction is performed by employing a mutation database obtained from UniProtKB/Swiss-Prot and other experimental data previously generated from mutagenesis experiments [18];
- MutationAssessor predicts the functional impact of amino acid substitutions on proteins using the evolutionary conservation of the affected amino acid in protein counterparts. Multiple Sequence Alignment is used to reflect functional specificity, represent the functional impact of a missense variant, and generate conservation scores. Variants with higher scores are more likely to be pathogenic [19];
- MutationTaster2 predicts functional changes in DNA sequences. It is designed to predict consequences based on amino acid substitutions, and intronic substitutions such as synonymous changes, short insertion or exclusion mutations, and variants that cover the limits of introns and exons [20];
- Likelihood Ratio Test (LRT) is a metric that evaluates the proportion of synonymous and nonsynonymous mutations in protein-coding regions. The altered proportion of mutations means that a negative selection process occurred over that region during evolution, which consequently modifies codons in peptide chains [21].

### 2.3. Encoding Genome-Wide Training and Test Dataset

After functional annotation, we preprocessed ClinVar data according to ACMG-AMP pathogenicity labels. In this step, we grouped these classes into labels: (a) benign/likely benign into benign; (b) pathogenic/likely pathogenic into pathogenic; (c) variant of uncertain significance and variant with a conflict of interpretation remained with the same label.

A second round of data preprocessing was performed for filtering ClinVar data to avoid variants with missing data. The training/test dataset comprised only variants that were classified by the eight aforementioned single predictors of functional impact. Following functional annotation, the ClinVar dataset was preprocessed using in-house scripts for data extraction and encoding methods. For this study, we investigate data-encoding strategies and representation-learning strategies:

- Label encoding is an approach that assigns numerical values from 0 to the number of classes −1 to each of the categorical values in a dataset. For example, if the column with categorical values contains five classes, then the label encoding assigns numerical values between 0 and 4;
- One hot encoding transforms categorical variables using a dummy strategy. Each variable category is transformed into a binary column. For example, given a dataset with two categories, the one hot encoder creates two new columns to store binary values, 0 or 1;
- Multiple Correspondence Analysis (MCA) is a statistical method that handles categorical variables for dimensionality reduction. MCA is an extension of simple correspondence analysis and a generalization of principal component analysis, which is appropriate for quantitative data [22]. The MCA is used to create a low-dimensional space for samples and predictor points based on a contingency table, and the dimensions are retained as eigenvalues;
- Autoencoders are unsupervised learning algorithms that aim to obtain a data representation by reconstructing the input data at the output [23]. In this study, artificial neural networks were implemented to learn representations of the ClinVar data. We used an autoencoder similar to a multilayer perceptron (MLP), with an input layer, a hidden layer with 10, 20, and 30 neurons, and an output layer with the same number of predictors. Rectifier (ReLu), Rectifier with Dropout, and Hyperbolic Tangent Function (Tanh) were used as neuron activation functions. Dropout is commonly used to reduce overfitting and can improve the results of a classifier. The function of this regularization layer is to turn off a portion of the neurons, forcing the network to readjust the weights and preventing the network from memorizing the training data [24].

### 2.4. Fine-Tuning of Random Forest

RF is a machine learning method created to avoid the limitations of single predictors, being an ensemble method that combines decision trees for classification or regression problems [25]. Essentially, each tree handles a subset of bootstrapped data from the original set of samples, as well a random subset of predictors [26]. This random sampling raises a low correlation between individual decision trees, which avoids overfitting. The prediction probability for each class is used to reach a final decision and take a majority vote.

We performed a grid-search strategy for fine-tuning RF models taking as input the categorical data, one hot encoded data, and representation-learned data extracted from MCA and autoencoders. The grid search strategy targets two RF parameters: (a) the number of trees in the forest model, that ranges from 50 to 1000 decision trees, and (b) the number of bootstrapped predictors (p), that was set to 2, $\sqrt{p}$, p/2, p. The parameter values were chosen based on experiments from [5,27]. Thus, we defined three experiments, as follows:

(1) RF were trained with categorical data and one hot encoder;
(2) RF were trained with two extracted MCA dimensions;
(3) RF were trained with two dimensions from autoencoders based on three diferent activation functions: rectifier, rectifier with dropout and tahn. Moreover, we range the number of epochs and hidden neurons on autoencoders, which were set for 10, 20, and 30 for both parameters.

For model evaluation, we considered the Area Under Curve (AUC) and the out-of-bag error (OOBE), a strategy similar to cross-validation [28]. AUC is derived from Receiver Operating Curves and represents the degree of class separability, in which values close to 1 represent high-grade model performance. All models were implemented using R base and *randomForest* (v.4.6-14) and *h20* (v.3.34.0.3) libraries.

### 2.5. AmazonForest: Web Platform for Variant Classification

We developed the online version of AmazonForest to improve user experience on pathogenicity prediction. AmazonForest was implemented as an online platform that performs our best metaprediction model to predict the pathogenicity of VUS, CI, and new genetic variants. AmazonForest is available at https://www2.lghm.ufpa.br/amazonforest, accessed on 6 February 2022. The platform is divided into two components:

- The first is the user interface component. AmazonForest was developed as a web tool with an interface that allows performing pathogenicity prediction of SNPs or INDELs with in silico analyses employing the best metapredictor model. The simple web interface enables the user to predict pathogenicity in two ways. First, by providing genomic or dbSNP information (chromosome, chromosome position, or rsID) and second, by allowing the combination of predictor results to query pathogenicity status. The web component was developed using Python3.6 [29], Javascript (https://developer.mozilla.org/en-US/docs/Web/JavaScript/Reference), HTML5 (https://developer.mozilla.org/pt-BR/docs/Web/HTML/HTML5), and using frameworks such as Flask (v.2) (https://palletsprojects.com/p/flask/), scikit-learn [30], Pandas (v.1.1.5) [31], Numpy (V.1.19.5) [32]. All packages was acessed on 2 February 2021.
- The second is a model administrator component to assess the evolution and performance of the model. This model component enables the reproducibility of up-to-date data.

## 3. Results

### 3.1. Training and Test Data Records

**Figure 1:**
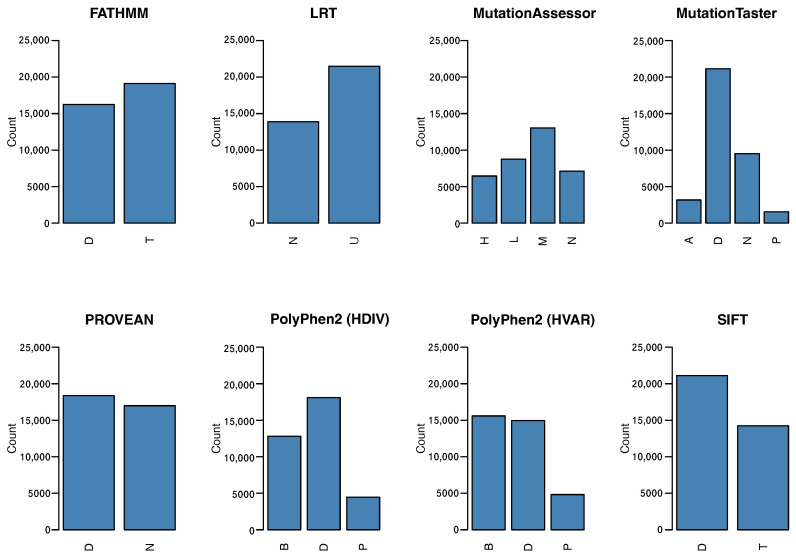
Distribution of variants by functional impact prediction for the eight predictors described in Section 2.2. Each functionial predictor provides their own type of classification. Deleterious (D) and Tolerated (T) for FATHMM; neutral (N) or unknown (U) for LRT; high (H), medium (M), low (L), or neutral for MutationAssessor; disease-causing, automatic prediction (A), disease-causing (D), probably harmless automatic prediction (N), and known to be harmless (P) for MutationTaster; deleterious (D) an neutral (N) for PROVEAN; probably damaging (D), possibly damaging (P) and benign for Polyphen; and finally, deleterious (D) and tolerated (T) for SIFT.

The filtering strategy for ClinVar’s database resulted in a slightly unbalanced training dataset without missing data, and more benign variants were cataloged than pathogenic variants. A view of the set of variants in this process is shown in Table 1, which highlights the original number of cataloged variants in the ClinVar database, the distribution of variants by class for the training/test dataset, and the reclassified dataset. Furthermore, data preprocessing showed a significant decrease in the number of genetic variants with functional annotation for each of the eight predictors. The distribution of categorical data was drawn in Figure 1, which highlights the challenge and complexity of interpreting the functional impact of variants. Additionally, we established the number of epochs and hidden neurons on autoencoders, which were set for 10, 20, and 30 for both parameters.

### 3.2. Fine-Tuning and Selection of Metaprediction Model

**Figure 2:**
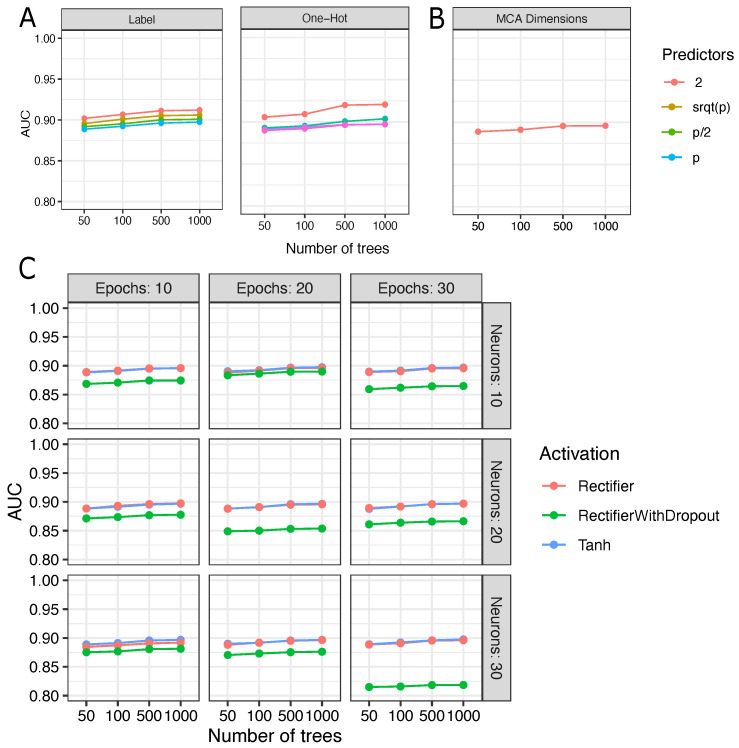
Fine-tuning analysis of Random Forest models. The Random Forest models were trained with label encoding and one hot encoding; learned data from multiple correspondence analysis and neural networks as autoencoders. (**A**) Random Forest shows high values of AUC when data is one hot encoded; (**B**) AUC results for Random Forest models trained with learned data from multiple correspondence analysis; (**C**) AUC results for Random forest models trained with autoencoded data.

RF training yielded 144 accurate models for variant pathogenicity prediction. All fine-tuning experiment results were drawn in Figure 2. In the experiments, AUC ranged from 0.88–0.91 using label encoding data, 0.88–0.92 when one hot encoded data were employed, 0.88–0.89, and 0.81–0.89 for representation learned data extracted from MCA and deep autoencoders, respectively. The best RF model reached higher AUC value with 1000 trees and two bootstrapped predictors under training. This model was trained with one hot encoded data and showed an AUC of 0.93 and an OOBE of 14.1% (see Figure 2A). For this model, feature importance analysis by Gini impurity (GI) identified PROVEAN and MutaTaster as the most influential features (GI > 0.2). In decreasing order (GI ≤ 0.1) of importance, GI identified PolyPhen_Hvar, SIFT, PolyPhen2_HDIV, FATHMM, LRT_pred, and MutaAss (see Supplementary Figure S1).

Extraction of representation-learned data from MCA and autoencoder models did not reach higher AUC combined with RF, but are satisfactory models. Compared with label encoding and one hot encoding, the RF model showed the lowest AUC when trained with representation-learned data from MCA or autoencoder data. RF trained with autoencoder data extracted from deep learning models, with Rectifier and Tanh activation functions performed similarly. Most of the AUC for these experiments overlapped (see Figure 2). In contrast, AUC is lower for all the experiments using RF models with autoencoder data from deep learning models trained with rectifiers with dropout. Additionally, we observed the lowest AUC for autoencoders set with rectifier with dropout, higher values in the number of hidden neurons, and trained with a higher number of epochs (see Figure 2C).

In addition to the aforementioned model comparisons, we compared RF, with Naive Bayes (NB), and Support Vector Machine, which showed satisfactory prediction performance, AUC >0.9 (see Supplementary Table S2 and Figure S2). All models were evaluated by performing 10-fold cross-validation. The SVM model trained with linear kernel showed similar results to RF (AUC = 0.93, +/−0.01). Based on this evaluation analysis and characteristics of RF and SVM, we chose RF for further analysis, given that SVM performes better on noncategorical data, has a costly computational complexity and high training time for large databases.

### 3.3. Reclassification of VUS and CI Variants

**Figure 3:**
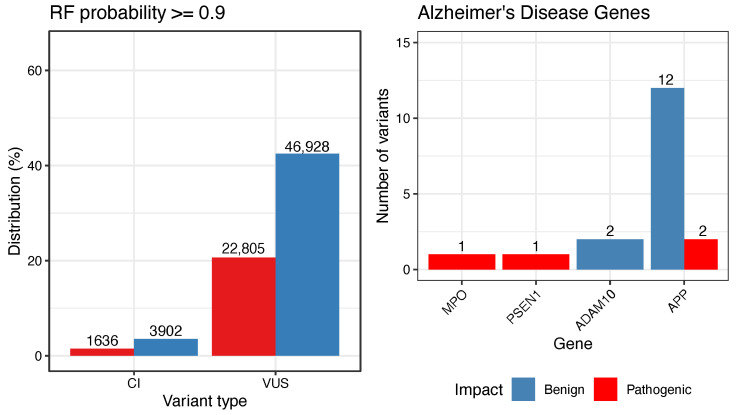
On the left, distribution of CI and VUS classified into benign and pathogenic after impact prediction with probability ≥0.9 by AmazonForest. On the right, distribution of variants for Alzheimer’s Disease-related genes.

The best RF model was applied to classify 100,805 genetic variants labeled as variants of uncertain significance or conflict of interest. As a result, 32,398 (32.14%) VUS and 2282 (2.26%) CI variants were labeled as pathogenic variants. Out of this last set, we identified a set of 24,428 genetic variants with high-probability of pathogenicity according to RF predictions ($RF_{prob}\geq 0.9$, see Figure 3A). These variants were distributed throughout 1019 gene regions. Reactome pathway analysis was performed for those genes, which revealed a set of 24 enriched pathways (Supplementary Table S1). The enriched pathways are associated with many important cell functions, such as metabolic processes, cell growth and division, extracellular matrix organization and degradation, muscle contraction, and cardiac conduction. Thus, missense variants related to these pathways may disrupt biological processes.

### 3.4. Case Study: Alzheimer’s Disease-Related Genes

Genetic studies have identified candidate disease genes by mapping SNPs that may contribute to the development of dementia traits, such as Alzheimer’s Disease (AD). Moreover, AD is a multifactorial and complex disease with a genetic basis that remains to be elucidated [6,33]. In ClinVar data, 18 SNPs (CI or VUS) are associated with AD. Prediction results show four pathogenic variants and 14 benign variants (see Figure 3B and Table 2). The Aβ precursor protein (APP) gene shows 13 VUS and one CI variant. Two variants in the APP region were predicted as pathogenic, which may impact protein structure (NM_000484.4, c.982C>T, p.Arg328Trp) and (c.298C>T, p.Arg100Trp). MPO showed one pathogenic variant (c.1031G>A, p.Gly344Asp), as well PSEN1 (c.475TC, p.Tyr159His).

Molecular interactions between the aforementioned genes have been associated with AD. Extracellular formation of senile plaques, which are insoluble deposits of neurotoxic amyloid-β (Aβ) peptides along with metal ions, is a histopathological hallmark of AD. Through redox reactions, metal ions are activated and may bond with Aβ to catalyze Reactive Oxygen Species (ROS) such as hydroxyl, a highly reactive radical. This reaction may induce inflammation and oxidative damage to surrounding molecules [34,35,36].

Myeloperoxidase (MPO) is a myeloid enzyme abundant in neutrophil granulocytes and monocytes but not detectable in microglia. It plays a primary role in inflammatory and degenerative processes [37,38]. Studies reported the presence of MPO levels in the frontal cortex in Aβ positive senile plaques and active microglia [38].

Mutations in presenilin-1 (PSEN1), presenilin-2 (PSEN2), and APP genes were previously described as a cause of autosomal-dominant early onset type AD [39,40] and familiar AD [41]. These genes are essential in the production of Aβ. APP encodes a precursor Aβ protein, which is processed by the β-secretase and the γ-secretase complexes and leads to the production of Aβ. PSEN1 and PSEN2 encode presenilins, which constitute the catalytic subunit of the γ-secretase complex [39]. PSEN1 is also reported to cleave another type I transmembrane substrate, which could negatively affect notch signaling [41].

Opposite to APP, PSEN1, and PSEN2 mechanisms, A Disintegrin And Metalloprotease 10 (ADAM10) reduces the formation of Aβ in physiological conditions and is associated with non-amyloidogenic and neuroprotective pathways [42]. ADAM10 encodes α-secretase, a protein complex which cleaves the Aβ region of APP, releasing a soluble fragment (sAPPα) [43]. Previous studies have reported neuroprotective properties of sAPPα and proposed its enhancement as a therapeutic strategy for AD and other neurodegenerative diseases [44].

## 4. Discussion

In this study, we evaluated the performance of RF trained with encoding data and representation learning extracted from MCA and neural network-based autoencoders, aiming to produce a metaprediction model (AmazonForest). The best RF model with one hot encoding was chosen for (re)classification of VUS and CI variants. This study is the first to investigate different encoding methods and influences on pathogenicity predictions by RF and representation learning algorithms. We found that encoding methods and autoencoders had little influence on RF models (see ROC and AUC in Figure 2).

Metaprediction approaches were proposed based on distinct machine learning or statistical methods and differ in training datasets [9,10,11,13]. In fact, most of the reviewed metapredictors adopted decision tree-based methods, [9,11,13], which deal with categorical predictors without the need to reconstruct them [45]. However, all metapredictors are unclear about how they handle missing data, which may produce biased models. To avoid missing data bias and to obtain a reliable and robust model, our study removed variants with missing data from the training set. Thus, VUS and CI variants were reclassified if they showed data for the aforementioned eight predictors.

Our proposed model was used for the pathogenicity prediction of VUS and CI variants. After prediction, we identified a valuable set of 24,428 variants, at a RF probability >= 0.9, identifying a variant dataset with a high probability of being pathogenic. This information could further improve our understanding of well-known diseases, as well as clarify molecular mechanisms involved in rare disorders. Therefore, AmazonForest can help to obtain more careful and accurate analyses of variants of uncertain significance and CI. Finally, we provided an online tool and well-annotated R scripts for a better user experience of pathogenicity prediction of genetic variants as well as (re)classification of CI and VUS variants.

The proposed model was compared to other prediction algorithms such as SVM and NB [46,47,48]. These additional comparison experiments are found in the Supplementary Materials.

## 5. Conclusions

Our benchmark shows that AmazonForest, a Random Forest-based model, presents satisfactory prediction results (AUC ≥ 0.93) regarding categorical data and one hot encoded data from eight functional impact predictors. Furthermore, we provide a new reclassified database and a model for programmatic prediction of large genetic variant sets of VUS and CI variants. Geneticists may consider the AmazonForest genetic variant data, and the web tool, for annotation of genome-wide studies, disease model tests, and investigations of variants pathogenicity and their associations to complex diseases, as demonstrated for Alzheimer’s disease.

## 6. Software Availability

AmazonForest is available online at: https://www2.lghm.ufpa.br/amazonforest. AmazonForest is constructed based on open source tools and all code is available at https://github.com/hpalheta/amazonforest. To use the metaprediction model we make availabe a R script, which are available on https://github.com/hpalheta/amazonforest/tree/master/meta_prediction/amazonforest.R. All data was accessed on 8 December 2021.

## Figures and Tables

**Table 1:** Distribution of genetic variants by functional impact in ClinVar original dataset. The training and test dataset is composed of biological annotated variants for the eight functional predictors described in Section 2.2.

Category of Genetic Variants in CinVar	Original Dataset	Training Dataset	Reclassification Dataset
Benign	266,145	18,891	-
Pathogenic	130,739	16,471	-
With conflit of interpretation	42,609	-	7193
With uncertain significance	349,926	-	93,612

**Table 2:** AmazonForest prediction results for reclassification of genetic variants in genes associated with Alzheimer’s disease.

Chromosome	Position	Gene	Protein	Protein Change	dbSNP ID	ClinVar Significance	AmazonForest Prediction
21	26000066	APP	NM_000484.4	c.982CT (p.Arg328Trp)		VUS	Pathogenic
21	26090000	APP	NM_000484.4	c.298CT (p.Arg100Trp)	rs200347552	VUS	Pathogenic
17	58278000	MPO	NM_000250.2	c.1031GA (p.Gly344Asp)		VUS	Pathogenic
14	73173702	PSEN1	NM_000021.4	c.475TC (p.Tyr159His)		VUS	Pathogenic
Chromosome	Position	Gene	Protein	Protein Change	dbSNP ID	ClinVar Significance	named-content content-type="color: #FFFFFF">AmazonForest Prediction
15	58665141	ADAM10	NM_001110.4	c.541AG (p.Arg181Gly)	rs145518263	VUS	Benign
15	58665172	ADAM10	NM_001110.4	c.510GC (p.Gln170His)	rs61751103	VUS	Benign
21	25997360	APP	NM_000484.4	c.1090CT (p.Leu364Phe)	rs749453173	VUS	Benign
21	25997413	APP	NM_000484.4	c.1037CA (p.Ser346Tyr)		VUS	Benign
21	26000018	APP	NM_000484.4	c.1030GA (p.Ala344Thr)	rs201045185	VUS	Benign
21	26000167	APP	NM_000484.4	c.881AG (p.Gln294Arg)		VUS	Benign
21	26021902	APP	NM_000484.4	c.803GA (p.Arg268Lys)	rs1601237753	VUS	Benign
21	26021954	APP	NM_000484.4	c.751GA (p.Gly251Ser		VUS	Benign
21	26021978	APP	NM_000484.4	c.727GA (p.Asp243Asn)		VUS	Benign
21	26022001	APP	NM_000484.4	c.704CT (p.Ala235Val)		CI	Benign
21	26022031	APP	NM_000484.4	c.674TC (p.Val225Ala)	rs746313873	VUS	Benign
21	26051060	APP	NM_000484.4	c.602CT (p.Ala201Val)	rs149995579	VUS	Benign
21	26051088	APP	NM_000484.4	c.574GA (p.Glu192Lys)		VUS	Benign
21	26170574	APP	NM_000484.4	c.47GA (p.Arg16Gln)		VUS	Benign

## Data Availability

The training/test dataset was made available at https://github.com/hpalheta/amazonforest/blob/master/meta_prediction/clinvar.train.csv. The reclassified variant dataset was made available at https://github.com/hpalheta/amazonforest/blob/master/meta_prediction/clinvar.civus_new_pred.csv, which can be loaded easily in R environment. All data and models was accessed on 8 December 2021.

## Supplementary Materials

The following are available online at https://www.mdpi.com/article/10.3390/biology11040538/s1, Figure S1: Gini impurity index for eight functional impact predictors; Figure S2: ROC curves for Naive Bayes, Random Forest and Support Vector Machine; Table S1: Reactome pathway enrichment analysis of genes mapped for VUS and CI genetic variants with pathogenicity probability equals to 0.9; Table S2: Accuracy, F1-score and mean AUC for Naive Bayes Random Forest and SVM.

## Abbreviations

The following abbreviations are used in this manuscript:

NGS	Next Generation Sequencing
GWAS	Genome Wide Association Studies
SNP	Single Nucleotide Polymorphism
ML	Machine Learning
VUS	Variants of uncertain significance
CI	Conflit of interpretation
RF	Random Forest
ROC	Receiver Operating Curve
AUC	Area Under Curve
