# Supplementary material for: AmazonForest: In Silico Metaprediction of Pathogenic Variants
Source: Biology (Basel). 2022 Mar 31;11(4):538. doi: 10.3390/biology11040538 (PMC9024711; doi:10.3390/biology11040538)
Supplement: Supplementary file 1 [file biology-11-00538-s001.zip › biology-1591898-supplementary.pdf]

biology

MDPI

Article

Supplementary Material - AmazonForest: In Silico Metaprediction of pathogenic variants

1. Feature Importance

Feature importance were accessed for the best Random Forest model combined with label encoder. The Figure S1 shows the distribution of Gini impurity index for the eight predictors.

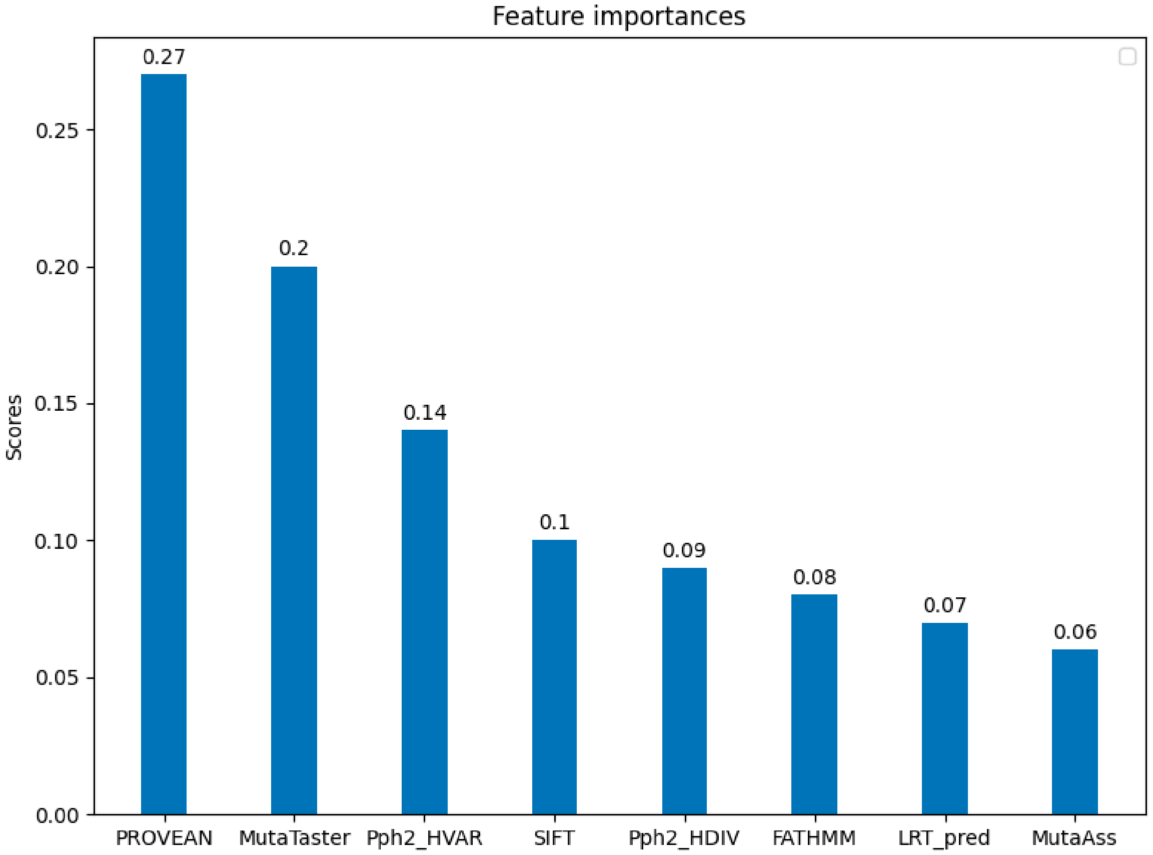

Figure S1. Gini impurity index for eight functional impact predictors.

2. Enriched Pathways

Biological Pathway	Genes	False–Discover Rate
Diseases of metabolism	76	3.19E-07
Extracellular matrix organization	56	1.40E-04
Muscle contraction	42	0.00200118
Degradation of the extracellular matrix	37	4.41E-06
Diseases of glycosylation	35	0.0048245
Cardiac conduction	30	0.00821261
Signaling by PDGF	26	1.15E-05
ECM proteoglycans	25	1.07E-05
Integrin cell surface interactions	25	4.56E-05

Collagen formation	25	4.92E-04
Collagen degradation	24	4.41E-06
Signaling by MET	23	3.34E-04
Collagen biosynthesis and modifying enzymes	22	1.40E-04
Assembly of collagen fibrils and other multimeric structures	21	9.67E-05
NCAM signaling for neurite out-growth	21	1.40E-04
Collagen chain trimerization	20	1.97E-06
Non-integrin membrane-ECM interactions	19	2.27E-04
NCAM1 interactions	16	2.27E-04
MET promotes cell motility	14	0.0035418
MET activates PTK2 signaling	13	5.12E-04
Laminin interactions	12	0.00177183
Signaling by PDGFRA extracellular domain mutants	9	0.00355751
Signaling by PDGFRA transmembrane, juxtamembrane and kinase domain mutants	9	0.00355751
Anchoring fibril formation	8	0.00399627

Table S1. Reactome pathway enrichment analysis of genes mapped for VUS and CI genetic variantswith pathogenicity probability equals to 0.9.

3. Performance of Naive Bayes, Random Forest and Support Vector Machines

The model evaluation of Random Forest models indicates stability concerning AUC values, close to 0.93, as shown in the main text (see, Section 3.2, Figure 2). In this way, we chose the RF model trained with one-hot to perform further analyses. For comparison purposes, we performed experiments with the Naïve Bayes method and Support Vector Machines.

Naive Bayes (NB) is a probabilistic classifier algorithm, which assumes that all features are independent, so one feature does not influence other [46]. NB is based on the Bayes Theorem. Thus a simplistic classifier that has been reported to have an excellent performance regarding execution time and accuracy, even when considering analyses of large datasets [47]. The NB finds the probability of a given set of predictors for all possible values of the class variable y and finds the maximum probability. This procedure can be expressed mathematically as: $y = argmax_y P(y) \prod^{n} P(x_i|y)$. In the experiment with NB, we kept the default parameters available in the scikit-learn library (https://scikit-learn.org/stable/modules/naive_bayes.html, accessed on 6 February 2022).

Support Vector Machine (SVM) is a machine learning method based on statistical learning theory.Proposed by Vapnik [48], SVM has been applied for classification and regression problems. SVM performs the classification of samples based on a set of hyperplanes estimated from high-dimensional datasets. If samples are not linearly separable, a nonlinear transformation is performed. SVM takes advantage of kernel functions for this purpose. Kernels such as the radial basis function, linear, polynomial, and sigmoid were used in the training step to select support vectors that outline the best hyperplane in the feature space. The implementation is available at https://scikit-learn.org/stable/modules/svm.html,accessed on 6 February 2022.

In general, the evaluation results of Random Forest, Naive Bayes, and SVM models indicate good performance (AUC > 0.9), as can be seen in Table 2 and Figure 2. However, the SVM model trained linear kernel yields similar results to Random Forest. The AUC for the two models corresponds to 0.93 with a standard deviation of 0.01. Thus, we chose Random Forest to compose the AmazonForest base, in contrast to the SVM, which

deals better with non-categorical data, has a costly computational complexity and training time for large databases.

Table S2. Accuracy, F1-score and mean AUC for Naive Bayes Random Forest and SVM.

Model	Accuracy	F1-Score	Mean AUC
Naive Bayes	0.844	0.840	0.92
Random Forest	0.861	0.852	0.93
SVM- RBF	0.858	0.850	0.90
SVM - Linear	0.861	0.854	0.93
SVM- Poly	0.856	0.849	0.91
SVM - Sigmoid	0.813	0.795	0.88

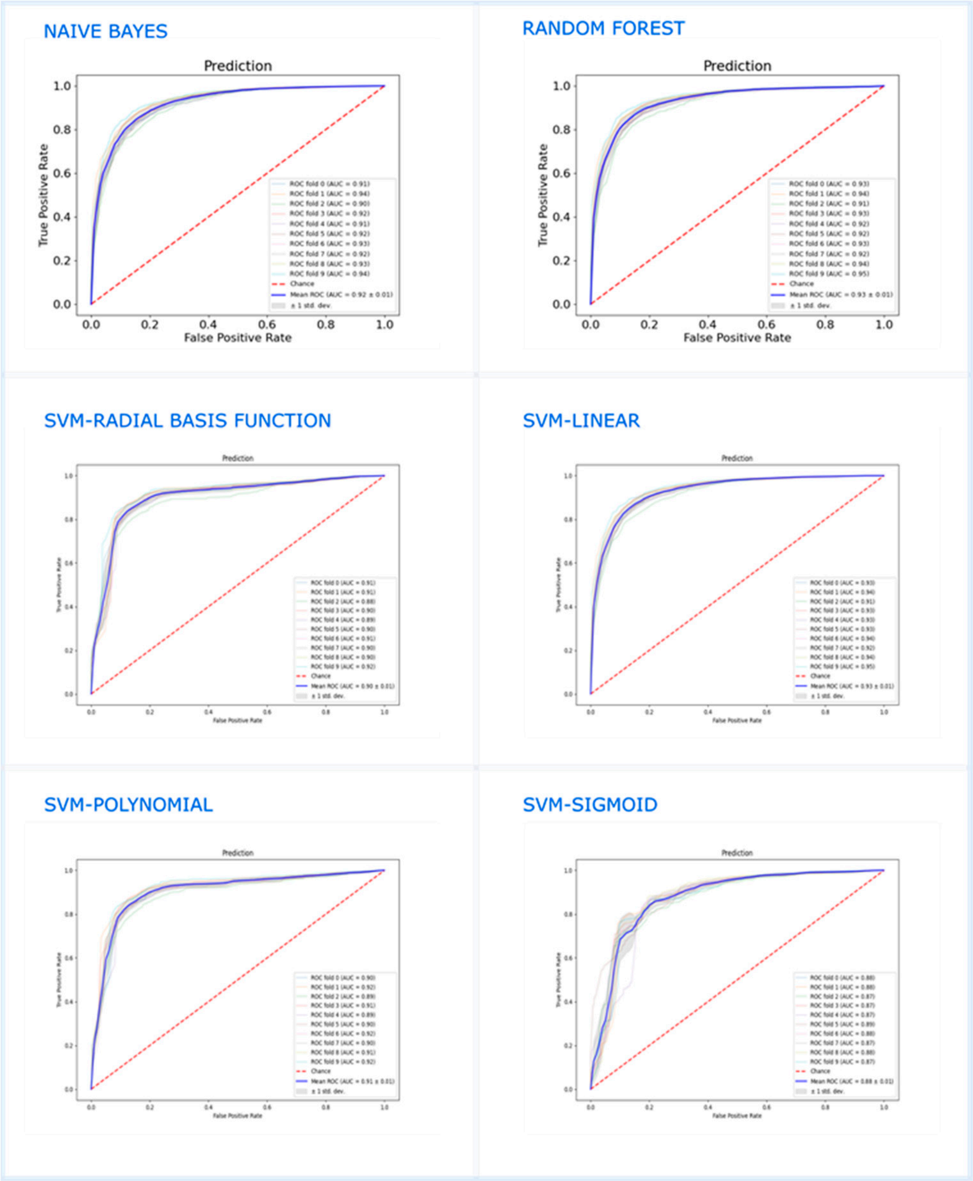

Figure S2. ROC curves for Naive Bayes, Random Forest and Support Vector Machine.

References

46. Mitchell, T.M. Generative and discriminative classifiers: Naive bayes and logistic regression. *Machine learning* **2010**, pp. 1–17.

47. Rish, I.; others. An empirical study of the naive Bayes classifier. IJCAI 2001 workshop on empirical methods in artificial intelligence, 2001, Vol. 3, pp. 41–46.

48. Cortes, C.; Vapnik, V. Support-vector networks. *Machine learning* **1995**, *20*, 273–297.
